# Natural Semantic Networks: The Concept of Mistreatment and Good Treatment in Students of Health Careers

**DOI:** 10.3390/bs14111072

**Published:** 2024-11-10

**Authors:** Javiera Ortega-Bastidas, Marjorie Baquedano-Rodríguez, Nancy Bastías-Vega, Cristhian Pérez-Villalobos, Mary Jane Schilling-Norman, Paula Parra-Ponce, Ricardo Arteaga-San Martín, Marcela Hechenleitner-Carvallo, María Isabel Ríos-Teillier, Ximena Paredes-Villarroel, José Peralta-Camposano, Alejandra Ricouz-Moya, Catherine Soto-Faúndes, Carolina Williams-Oyarce

**Affiliations:** 1 Medical Educational Department, Faculty of Medicine, Universidad de Concepcion, Chacabuco Esquina Janequeo s/n, Concepción 4030000, Chile; nbastias@udec.cl (N.B.-V.); cperezv@udec.cl (C.P.-V.); maryschilling@udec.cl (M.J.S.-N.); paulaparra@udec.cl (P.P.-P.); 2 Department of Economics and Finance, Faculty of Business Sciences, Universidad del Bío-Bío, Avenida Collao 1202, Concepción 4030000, Chile; mbaquedano@ubiobio.cl; 3 Institute of Movement Sciences and Human Occupation, Faculty of Medicine, Universidad Austral de Chile, Independencia 631, Valdivia 5110566, Chile; ricardo.arteaga@uach.cl; 4 Department of Basic and Morphological Sciences, Office of Health Sciences Education, Faculty of Medicine, Universidad Católica de la Santísima Concepción, Alonso de Ribera 2850, Concepción 4090541, Chile; marcelahc@ucsc.cl; 5 Department of Clinical, Faculty of Medicine, Universidad Católica del Norte, Larrondo 1281, Coquimbo 1780000, Chile; mrios@ucn.cl; 6 Department of Health Sciences, Universidad de Aysén, Eusebio Lillo 667, Coyhaique 5950000, Chile; ximena.paredes@uaysen.cl; 7 Department of Health Sciences Education, Faculty of Medicine, Universidad de Chile, Avenida Independencia 1027, Santiago 8380000, Chile; jperalta@uchile.cl; 8 Coordination of Educational Management in Health, Department of Health, Universidad de Los Lagos, Avenida Alberto Hertha Fuchslocher 1305, Osorno 5290000, Chile; alejandra.ricouz@ulagos.cl; 9 School of Medicine, Faculty of Medicine, Universidad de Valparaíso, Angamos 655, Viña del Mar 2520000, Chile; catherine.soto@uv.cl; 10 Office of Medical Education and Health Sciences, School of Medicine and Health Sciences, Universidad Central de Chile, Lord Cochrane 417, Santiago 8320000, Chile; carolina.williams@ucentral.cl

**Keywords:** mistreatment, good treatment, health careers, natural semantic network

## Abstract

Natural semantic networks (NSNs) provide an approach to understanding concepts in terms of their density and strength. The objective of this study was to conceptualize mistreatment and good treatment among health science students in Chile, according to gender and training cycle. **Methods**: A mixed-methods study with a relational scope was conducted, involving 994 health science students. The present study is exploratory and combines both qualitative and quantitative analysis. We utilized the NSN technique, applying a questionnaire via SurveyMonkey after obtaining informed consent. **Results**: A total of 4386 defining words for mistreatment were identified, with the most relevant being ‘aggression’, ‘abuse’, and ‘violence’. In the case of good treatment, a total of 4146 defining words were obtained, with ‘respect’, ‘empathy’, and ‘kindness’ standing out. For good treatment, a statistically significant relationship was observed between the defining words and the variables of gender (χ^2^ = 41.158; *p* < 0.05) and training cycle (χ^2^ = 28.698; *p* < 0.05). For mistreatment, a statistically significant relationship was found only with training cycle (χ^2^ = 35.858; *p* < 0.05). **Conclusions**: Exploring the meanings associated with mistreatment and good treatment has a theoretical implication in the way in which two highly polysemic aspects of the educational environment can be understood.

## 1. Introduction

One of the virtues we can highlight from the educational process is its transformative potential. It allows individuals to venture into a practical field where they will deploy their own skills and knowledge. These opportunities enable individuals not only to insert themselves into a specific labor market but also to identify with a particular role. In the health field, this professional and personal combination becomes especially relevant, particularly when educational contexts manage to balance clinical teaching in an environment conducive to integrating both aspects. In this regard, it is important to emphasize that the experience from which learning is developed is accompanied by the modeling of the teacher, and the interaction that arises from teaching is essential to achieving a positive internalization of it [1,2,3]. Furthermore, educational research has emphasized that environments that foster meaningful learning are those that effectively promote students’ well-being and mental health [4], which later enables them to fully perform in their professional role [5]. The problem arises when these instances are tainted by environments that do not favor students’ proper development or are undermined by situations where humiliation, belittlement, harassment prevail, or where the hidden curriculum and professional habitus become constituents into a cultural of violence [6,7]. Positive educational spaces for learning and those that create harmful environments essentially act as accents of the same phenomenon: the educational environment.

**What evidence exists of mistreatment in health careers?**

Mistreatment is a phenomenon that has been visible for decades, defined as an intentional or unintentional event that occurs when behavior shows disrespect for the dignity of others and unreasonably interferes with the learning process [8].

In 1982, Silver emphasized that some of the consequences for students included discouragement, depression, fear, and frustration [9]. In this regard, Silver raised two questions: Was abuse among medical students a common occurrence? Was it now necessary to determine the causes, frequency, treatment, and prevention of mistreatment? [9]. A field of discussion was then opened where researchers had to sharpen their focus to elucidate the behavior of this phenomenon in the training process and to warn about the consequences it would have for professional experience. Today, various evidence highlights the consequences of mistreatment in education, particularly by clinical staff or teachers [10]. The findings of Hayward et al. [11] suggest that the mistreatment of medical students, and even the fear of future mistreatment, can negatively impact the open communication between medical students and the rest of the health care team, as well as their perceptions of assessment and their potential professional options. Additionally, students who perceive a negative learning environment are more likely to develop higher levels of burnout and lower levels of empathy [12]. These situations are observed among students in various health professions such as nursing [13], midwifery [14,15] and dentistry [16,17]. In specialty programs, Lall et al. examined the prevalence, types, and sources of perceived mistreatment in the workplace and the association between mistreatment and suicidal ideation [18].

In Latin America, it has been observed that mistreatment in the training of health students is frequent, as shown by studies in Colombia [19], Peru [20,21], and Paraguay [22]. In Chile, for example, it was found that 98.11% of undergraduate medical students had experienced mistreatment, a percentage higher than that evidenced in other studies [23]. Therefore, there is sufficient data indicating the need for urgent action to protect the physical and emotional integrity of students [24]. All this requires, first, a clear conceptualization of mistreatment, especially if the aim is to promote protocols or policies in higher education institutions.

The literature is extensive in this regard, as there are definitions that include dimensions of psychological violence, harassment, humiliation, defamation, belittlement, or verbal aggression, as well as physical violence that endangers individuals’ integrity [24]. There are also connotations in the academic realm, such as demoralization and unregulated demands [23]. Galtung, for his part, broadens the notion that violence operates in an interrelated manner, ranging from the explicit manifestation of direct violence to the legitimization of its use in cultural and structural terms [25]. It is then possible to speak of an abusive culture [26], which can be approached from its subjective dimension. In other words, violence must be situated from a multidimensional perspective, considering social, historical, and cultural aspects that help, in the words of Hernández and Alvear, “to highlight the elements that make it possible” [7] (p. 54). The perception associated with mistreatment may be broader than we can recognize or measure [27], which leads us to believe that there is still a need to conceptually investigate this phenomenon.

**What evidence exists of good treatment in health careers?**

Although the concept of good treatment has not been widely defined in the scientific literature, Dois and Bravo [3] situate it as an ideal condition in the clinical relationship, linked to the right to health, respect, and a possibility for the teacher to define themselves in the execution of their role, which leads to promoting a fair, empathetic evaluation and being aware of the competencies they model in direct interaction with the health user. In this way, good treatment would prefigure a positive mode of the educational relationship. To elucidate this in detail, it is necessary to unify the different dimensions that could account for this phenomenon. Why? It is not surprising that the evaluation of the educational environment has become necessary, precisely because it allows for recognizing students’ perceptions of the training processes in which they participate and the emotions they generate [28,29].

Considering the above, one of the reasons to situate good treatment as an ideal condition for the positive perception of the training experience in health is the intimate relationship between teacher models and academic well-being. A better perception of the educational environment is directly related to a better level of mental well-being [4]. Moreover, the interest in evaluating educational environments leads to recognizing a given training environment and, additionally, providing a complete overview of what happens in them [30]. For de Schwitz, Torti, and Lingard [31], the issue of well-being is a persistent reality in health training, and one of the needs is to clarify its conceptualization because there is a possibility of attributing individual responsibility to the educational experience, as is the case with attrition. Hanco-Monroy et al. [32] emphasize that the variables correlated with a lower risk of dropout are lifelong learning skills and subjective well-being. Thus, one could argue that good treatment constitutes a priori condition for academic well-being.

What should be questioned is whether subjective well-being in health training depends exclusively on personal disposition or whether it is shaped by relational factors. We prefer to highlight the second point, as the disposition and ability to establish empathetic, respectful, and caring relationships create an appropriate scenario for teachers and students to interact positively [33]. Along these lines, Sandoval, Dorner, and Véliz [34] observed how well-being is constructed through the subject’s ability to relate to their academic environment. This could support the fact that health training does not depend solely on the will of individuals but is also influenced by the circumstances in which the training experience takes place.

Another reason to consider recognizing good treatment as an ideal condition for a positive educational environment is the teaching style or the teacher’s role model. It has been shown that academic satisfaction is favored by constructivist pedagogical practices [35]. However, the distinctive feature of an effective teacher is their ability to communicate knowledge to students in a comprehensible and impactful way [1]. Ranjan et al. [2] observed significant variations in teaching styles depending on the level of teaching experience, gender, and institutions, but how the teacher presents themselves to the students is a fundamental aspect of teaching. What is at stake here is the teacher’s disposition to interact with their students to facilitate their training experience. This becomes relevant as students approach clinical years, since there is a sudden transition between teaching models due to the way clinical training is delivered and the complexities this level of training entails in practice [36,37].

Another domain that we could theoretically relate to good treatment is professionalism, as it is considered a value that enhances excellence, humanism, responsibility, altruism, and empathy [38]. Furthermore, it has been shown that professionalism predicts subjective well-being in health care professionals, especially when they are associated with skills in interprofessional collaboration, empathy, and lifelong learning [39]. At its core, professionalism permeates the entire curricular experience. During the clinical cycle, students observe experts in their activities and reasoning, the professional model, and their behavior with patients [36].

The growing evidence of the positive effects of good treatment in health careers underscore its importance not only for students’ emotional well-being but also for their professional competence and academic success. Recent research indicates that environments characterized by respect, empathy, and fairness not only foster greater student satisfaction but also improve the retention of essential clinical knowledge and skills [40]. These positive learning environments, according to Suikkala, Timonen, and Leino-Kilpi [41], are significantly associated with a lower incidence of medical errors in clinical simulations and real-world practices, highlighting how the treatment received during training can have direct implications for patient safety and the quality of medical care.

Therefore, it is imperative that educational institutions implement and maintain practices that promote good treatment, not only as an ethical issue in education but as a critical component of medical training that directly influences clinical competence, professional efficacy, and respectful patient care. Studies addressing positive aspects of the teacher–student relationship, a good educational environment, and academic well-being provide insights into domains that could establish good treatment as an ideal condition for a positive educational experience. It would then be no surprise that educational institutions have begun creating courses that promote social skills in students or, more specifically, communication skills [42].

**Why conceptualize mistreatment and good treatment in health careers?**

While there is extensive evidence on the importance of addressing the educational environment in health training, whether from a positive or negative perspective, there is still no specific conceptual definition of mistreatment and good treatment from the perspective of health science students. A conceptual approach that captures the meanings associated with these terms from the students’ perspective could broaden the ways in which research on this topic is approached. This would make it possible to include these dimensions in university evaluation systems, especially considering that there is currently greater awareness of students’ rights, which requires clinical training to continually seek ways to improve teaching, professional behavior, and the evaluation of these practices in their daily routine [5,43,44].

In the United States, the Association of American Medical Colleges (AAMC) created the Graduate Questionnaire (GQ) in 1978 as an evaluation system for medical schools and other organizations to identify and address critical issues for the future of medical education and students’ well-being [8]. Some of the areas they evaluate include student satisfaction, career and specialty plans, medical education costs, and students’ experiences of mistreatment in the learning environment [8]. In Chile, there are no national evaluation systems to assess the educational environment or determine whether experiences of mistreatment or good treatment exist in undergraduate or specialty training in various health careers. Evaluation systems that consider clear conceptualizations could be relevant for diagnosis and practical approaches.

Moreover, it is important to note that there is a common interest in educational studies that permeates the approach to the research itself, mobilizing the efforts given to them, which is to identify the ideal conditions for the positive development of the educational experience. In such a scenario, it is necessary to question the meanings that underpin concepts like mistreatment and good treatment to provide certain guidelines in terms of prevention and promotion in different educational cultures. The educational process constitutes a field of action for the formation of personal identity, and it is, therefore, necessary to have some clarity about the connotations that students themselves might give to such controversial concepts as mistreatment and good treatment.

Considering this, the present study aims to highlight two accents of the educational environment, which constitute a particular field of action: mistreatment and good treatment (Translation decisions: The concept of “mistreatment” is a direct translation of the Spanish concept “maltrato”. This translation is widely used in both Spanish and English scientific literature; therefore, we chose to follow the same logic. “Good treatment”, on the other hand, comes from the Spanish “buen trato”. Although it has not been widely used in the literature, we prefer this translation as it is more direct to the original concept. In English, there is no prefix—like “mis-” in “mistreatment”—that could be applied to this concept. While we could have used prefixes like “pro-” or “well-”, we believe they do not capture the original meaning of “buen” in Spanish). We will first develop a framework to assess the importance of both accents in the educational context, highlighting their impact on students’ experiences and the quality of educational training. In this way, we will highlight the existing evidence on good treatment and mistreatment in health sciences, analyzing studies that document examples of both positive and negative practices and their effects on the educational environment and students’ professional development. Considering this, the present research aims to conceptualize the meanings associated with mistreatment and good treatment from the perspective of health science students.

## 2. Materials and Methods

### 2.1. Study Design

The present study is exploratory with a relational scope and is based on a mixed methodology, combining both qualitative and quantitative analysis [45]. The research is part of a project funded by National Agency for Research and Development (ANID), Chile, ANID-Fondecyt No 1221913. As it is educational research, the guidelines provided by Chile’s National Regulations associated with scientific research involving people were followed: Law Nº 19.628 and Law Nº 20.120. Data collection was carried out after obtaining institutional authorization from each educational institution and informed consent in all cases.

The methodology used to collect and analyze the data in this research aims to capture a subjective domain of mistreatment and good treatment. Indeed, there is a relationship between the individual and their own subjectivity, which is expressed through various significant elements that give meaning to the individual’s lived experience. For Valdez, this denotes a particular issue of psychological meaning, which constitutes itself as a natural unit more “like an information code relative to a specific object” [46] (p. 13). The relationship the individual establishes with their own subjective world is full of meanings, and these can be elucidated through association networks that individuals respond to from certain given stimuli.

### 2.2. Study Setting

The study was conducted among undergraduate health science students from 9 Chilean universities. This setting was chosen because it allows for a comprehensive examination of the experiences and perceptions of future health care professionals in a diverse range of academic environments. By focusing on health science students, the research aims to capture the unique dynamics and challenges faced by this population, providing valuable insights into their understanding of good treatment and mistreatment in health care contexts.

This study involved 994 students from 12 health careers at 9 Chilean universities: nursing (22.5%; n = 224), speech therapy (2.4%; n = 24), physical therapy (10.3%; n = 102), medicine (41%; n = 408), nutrition and dietetics (7.7%; n = 77), midwifery and childcare (6.9%; n = 69), psychology (2%; n = 20), dentistry (1.6%; n = 16), chemistry and pharmacy (0.4%; n = 4), medical technology (4.1%; n = 41), occupational therapy (0.7%; n = 7), and biochemistry (0.2%; n = 2).

### 2.3. Study Duration and Validation

The study duration was set for one year (2023), during which data collection was carried out through an online survey. This timeframe was essential to ensure adequate participant recruitment and engagement across the nine universities involved in the study. Direct contact was established with various academic units to disseminate information about the research effectively, which facilitated a broader reach and encouraged participation among undergraduate health science students. The extended duration allowed for a thorough analysis of the collected data, contributing to the robustness and validity of the findings.

### 2.4. Sample Size

The sampling approach was carefully designed for methodological coherence. We applied a non-probabilistic quota sampling method with proportional allocation, using university affiliation and level of study as stratification variables. Given a small effect size (f = 0.1), a 95% confidence interval (1-α), and 80% power (1-β), we calculated that a sample of 994 students in health-related fields would adequately support our study’s objectives.

The inclusion criteria specified that participants must be regular students enrolled in health programs at 9 Chilean universities. The exclusion criterion was set as students who had been absent for more than two months in each evaluated semester, regardless of the reason. The selection of 12 health-related fields ensured diversity across professional perspectives, enriching our mixed-methods design with a broad spectrum of insights. Additionally, the inclusion of students from 9 universities provided a range of institutional and geographical contexts, enhancing external validity and supporting the study’s relational scope. Together, these sampling choices align with the study’s exploratory design, ensuring robust data for both quantitative and qualitative analyses.

### 2.5. Data Collection Tool and Study Variables

The instrument used was constructed using the Natural Semantic Networks (NSN) technique (México) [47], processed using the SPSS statistical package, version 24.0. This technique is relevant for exploring the meanings associated with concepts such as mistreatment and good treatment. The analysis consisted of two sections in which subjects had to indicate the defining word they considered most relevant to “Mistreatment” and “Good Treatment”. First, they had to define the concept by stating verbs, adverbs, adjectives, nouns, or pronouns without using articles or prepositions. Second, participants had to rank the relevance of each defining word they mentioned in the previous section. Before data cleaning, relationships of synonymy between words were analyzed using a double-entry matrix. This procedure is suggested to compact the information obtained from the original NSN technique and to avoid losing important information that may be representative in terms of word frequency and hierarchical relevance [46].

### 2.6. Data Collection Procedure

First, institutional authorization was obtained from the 9 participating universities across Chile. The focus of the study was to develop solutions by clarifying the concepts of mistreatment and good treatment in the context of health education. Subsequently, the heads of the health programs at these universities were contacted to explain the nature of the study and to organize the data collection processes. A survey was sent via SurveyMonkey to the program directors for dissemination to students, facilitating interest and participation within the university community. Efforts were made to promote the study’s purpose, emphasizing its national scope.

Data collection was conducted only after receiving institutional approval and obtaining informed consent from all participants. These processes detailed the study’s objectives, potential uses, types of participation requested, associated risks, and guarantees of freedom and voluntariness. Participants were assured of the confidentiality of their involvement and informed of their right to withdraw at any time. Additionally, the process of anonymization was explained to participants in the case of surveys.

### 2.7. Ethical Considerations

Participants were informed about the ethical aspects of the study through a detailed consent form. The study posed no anticipated physical risks; however, participants were informed that it might prompt reflections on their educational experience, potentially impacting their perceptions and satisfaction with their training. To address this, researchers committed to providing additional socio-emotional support opportunities at no cost. This study aims to identify challenges in health sciences education, specifically to conceptualize mistreatment and positive interactions from the students’ perspectives. Findings will be used to guide the design of protocols in participating universities to address these issues. All data provided by participants were secured through coded identification. Although participants’ IDs were initially collected for data organization, identifiers were replaced with unique codes before data storage to ensure anonymity. Only the lead researcher had access to the coding system, and participant identities were excluded from all analyses. Data were securely stored on password-protected devices, accessible solely by the research team and, if deemed necessary, by the reviewing Ethical-Scientific Committee.

### 2.8. Analysis Plan

A descriptive table was then constructed with all the defining words mentioned by the participants, and the main values of the semantic network were calculated based on the proposals of Figueroa, González, and Solís [47] and Valdez [46]. The values considered in the semantic network were as follows: (a) J-value, representing the total number of defining words mentioned by the participants. This value is an indicator of the semantic richness of the network, as a greater number of words indicates greater richness. This value was obtained by adding the total number of defining words attributed by the participants. (b) M-value, related to the hierarchy of each defining word. To obtain this value, the frequency of occurrence of the defining words was multiplied by the hierarchy assigned to each of them. The total M-values form the SAM set (Semantic Association Memory), which constitutes the central core of the semantic network, that is, the center of meanings related to the stimulus concept. Although the SAM set usually consists of the ten defining words with the highest M-value in the network that emerges from the data collection process, we selected the 14 words with the highest semantic weight. (c) Finally, the FMG-value was obtained, which refers to the semantic distance between the different defining words that make up the SAM set. The analysis was performed using the rule of three, starting with the defining words with the highest M-value in the network, which represents 100% in terms of percentage. For each defining word, the M-value was calculated by multiplying by 100 and dividing by the highest M-value in the network [46].

While the SAM set can serve as the basis for subsequent intra- and inter-group comparisons and correlations [47], for this study, we followed Hinojosa’s [48] considerations, using first-hierarchy frequencies as the main contrast variable. Pearson’s chi-square test was used to compare the defining words obtained in the first hierarchy with the variables of sex and training cycle. Additionally, a thematic analysis was conducted to categorize the defining words to highlight their bidimensional nature [49].

## 3. Results

**Figure 1:**
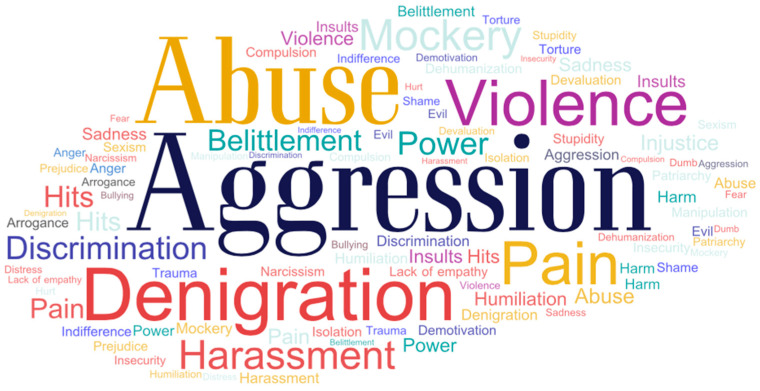
Visual representation of the J-value for mistreatment with a total of 4386 words.

Of the total participants, 29.8% (n = 297) were men, 69.3% (n = 689) were women, and 0.8% (n = 8) identified with another gender, with an average age of 22.4 (SD = 3.7). Regarding the training cycle, it was subdivided into two cycles: 39.8% (n = 396) were in the pre-clinical cycle, and 60.2% (n = 598) were in the clinical cycle. First, the defining words that emerged from the network of meanings provided by all participants were examined. As shown in Figure 1, a total of 4386 defining words were obtained from the mistreatment survey.

From these, 14 defining words can be identified that represent the strongest semantic weight for the concept of mistreatment. The general semantic core was mainly composed of the word ‘aggression’ (SAM = 2595), followed by ‘abuse’ (SAM = 2520), ‘violence’ (SAM = 2326), ‘humiliation’ (SAM = 1990), ‘harm’ (SAM = 1624), and finally, ‘belittlement’ (SAM = 1103). This primary set also includes the words ‘hits’ (SAM = 839), ‘insults’ (SAM = 757), ‘discrimination’ (SAM = 647), and ‘harassment’ (SAM = 647). Lastly, four defining words of lesser frequency compared to the previously mentioned words are observed: ‘mockery’ (SAM = 566), ‘pain’ (SAM = 564), ‘denigration’ (SAM = 564), and ‘power’ (SAM = 480). Table 1 identifies the frequency details of each defining word for the general semantic network of mistreatment, as well as the frequency of distance (FMG) that exists between each word.

**Figure 2:**
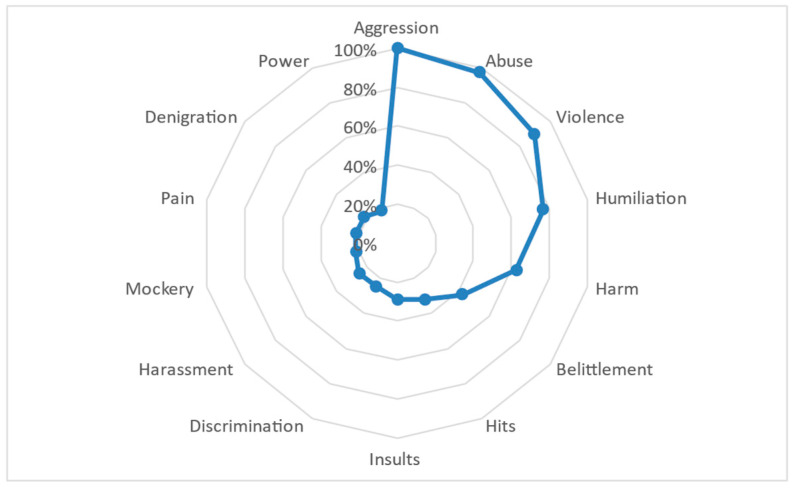
FMG distribution for the semantic network corresponding to the general SAM set for mistreatment.

Regarding the semantic distance between the 14 defining words (FMG), a slight distance is evident in the first subgroup of words in relation to the central concept of ‘aggression’, as represented in Figure 2. The words ‘abuse’ and ‘violence’ have a close proximity, indicating their strength in conceptualizing mistreatment, but they also have a broader connotation of the concept. Words such as ‘humiliation’, ‘harm’ and ‘belittlement’ have a more specific reference to behaviors that are visible in concrete actions. Moreover, greater distance is observed with words like ‘hits’, ‘insults’, ‘discrimination’, ‘harassment’, and ‘mockery’, but all continue to allude to specific behaviors, indicating that mistreatment is perceived through observable actions that one individual commits against another.

**Figure 3:**
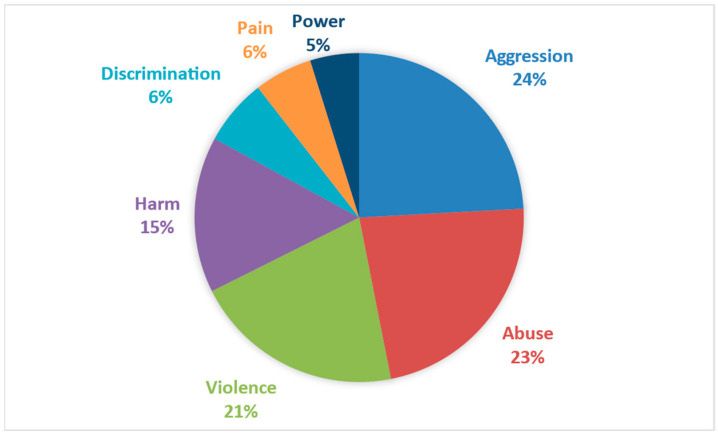
Distribution of the latent dimension associated with mistreatment.

Based on the above, the defining words for mistreatment were categorized according to their latent and explicit conceptualization. Regarding the latent conceptualization, the words associated with mistreatment refer to a broad dimension of the concept, which is not visible in specific behaviors. It refers to a latent dimension of it. In essence, this dimensionality helps describe the intangible aspects of mistreatment, as shown in Figure 3, ranging from words like ‘aggression’ to concepts like ‘power’.

**Figure 4:**
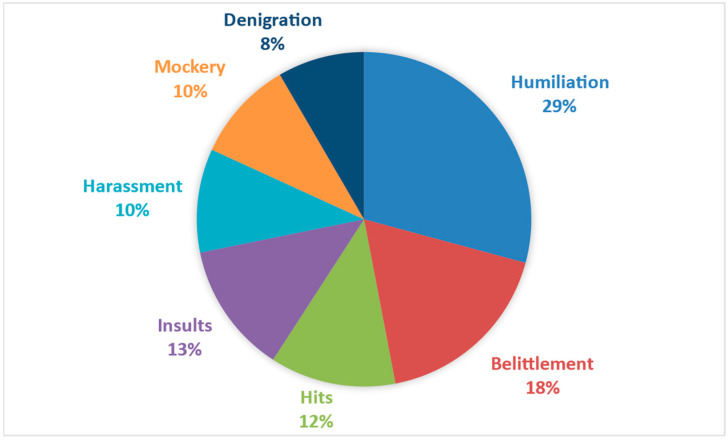
Distribution of the explicit dimension associated with mistreatment.

Regarding the explicit conceptualization, the words associated with mistreatment refer to observable behaviors and, therefore, can be identified in the specific context in which they occur. Thus, in Figure 4, it is possible to identify concepts such as ‘humiliation’, ‘belittlement’, or ‘denigration’, which contribute to a specific conceptualization of actions one individual can perform against another.

Second, the relationship between the defining words obtained in the first hierarchy of the SAM set and the variables of gender and training cycle (pre-clinical and clinical) was evaluated using Pearson’s chi-square test. Based on a bilateral contrast, it is evident that there is a statistically significant relationship between the defining words for mistreatment and the training cycle, x^2^ (35.858) = *p* < 0.05. In Table 2, the percentage frequencies obtained for each training cycle can be observed, and it is possible to recognize that the three words with the highest semantic weight that constitute the network of the latent dimensions have higher frequencies than the words associated with the explicit dimension.

**Figure 5:**
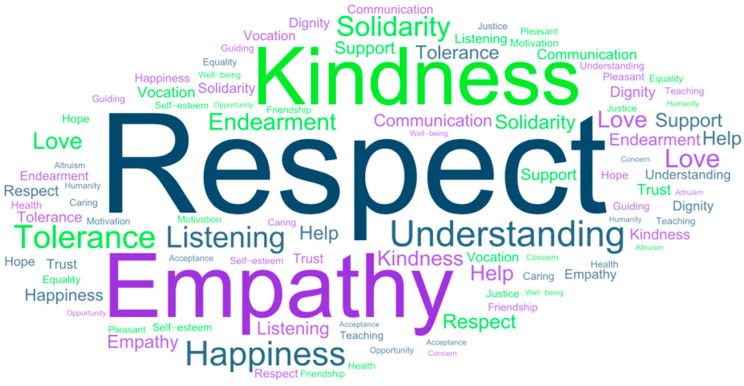
Visual representation of the J-value for good treatment with a total of 4146 words.

Third, the defining words that emerged from the network of meanings provided by all participants for the word good treatment were examined. As shown in Figure 5, a total of 4146 defining words were obtained.

From these, 14 defining words can be identified that represent the strongest semantic weight for the concept of good treatment. The general semantic core was mainly composed of the word ‘respect’ (SAM = 4319), followed by ‘empathy’ (SAM = 3861), ‘kindness’ (SAM = 1963), and ‘understanding’ (SAM = 1158). Accompanying this primary set were the words ‘happiness’ (SAM = 596), ‘tolerance’ (SAM = 593), ‘listening’ (SAM = 563), and ‘solidarity’ (SAM = 553). In a third group, with lower frequencies than the previously mentioned words, six relevant defining words were observed: ‘love’ (SAM = 492), ‘endearment’ (SAM = 480), ‘help’ (SAM = 455), ‘support’ (SAM = 447), ‘communication’ (SAM = 436), and ‘trust’ (SAM = 435). Table 3 details the frequency of each defining word for the general semantic network of good treatment, as well as the frequency of distance (FMG) that exists between each word.

**Figure 6:**
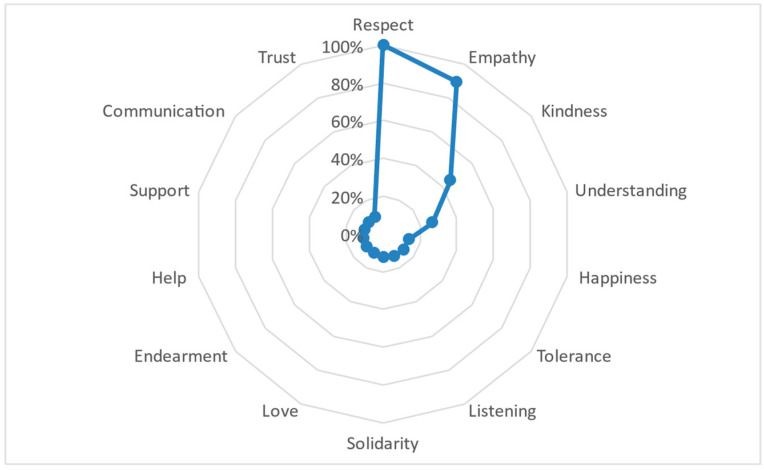
FMG distribution for the semantic network corresponding to the general SAM set for good treatment.

Regarding the semantic distance between the 14 defining words (FMG), a slight distance is evident in the first subgroup of words in relation to the central concept of ‘respect’, as represented in Figure 6. The word ‘empathy’ has a proximity, followed by a greater distance from defining words like ‘kindness’, ‘understanding’, and ‘happiness’. The distance observed with the remaining defining words is substantial but forms a particular subgroup of behaviors, such as ‘tolerance’, ‘listening’, ‘help’, ‘support’, and ‘communication’. This subgroup also includes words with a more abstract semantic order, such as ‘love’, ‘endearment’ and ‘trust’.

**Figure 7:**
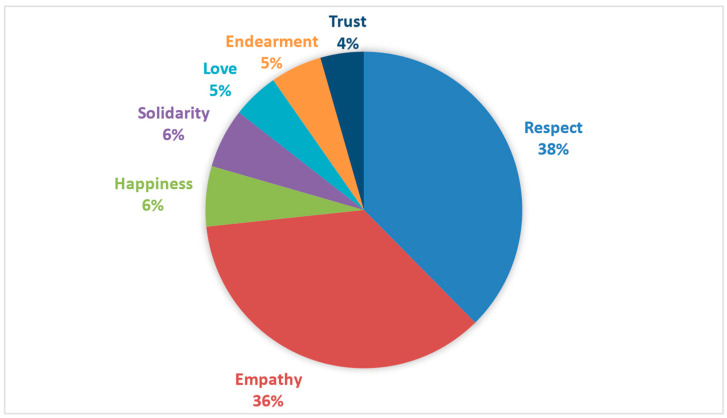
Distribution of the latent dimension associated with good treatment.

Based on the above, the defining words for good treatment were categorized according to their latent and explicit conceptualization. Regarding the latent conceptualization, the words associated with good treatment refer to ideal domains of the concept, denoting a mode of being in the educational relationship. In essence, this dimensionality helps describe the implicit aspects of good treatment, as shown in Figure 7, ranging from words like ‘respect’ to concepts like ‘trust’.

**Figure 8:**
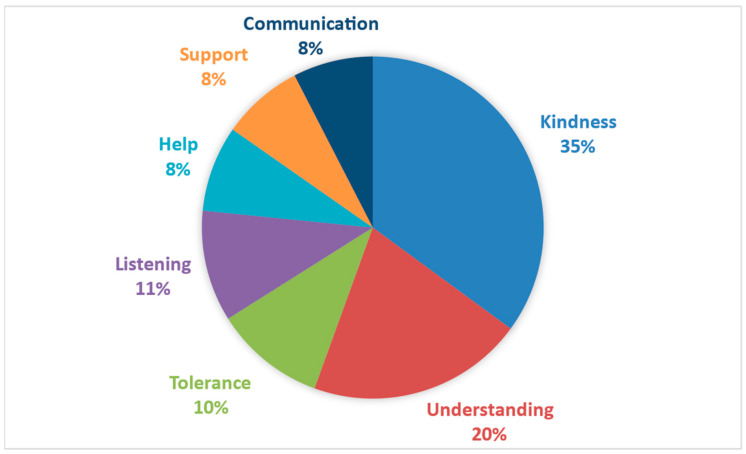
Distribution of the explicit dimension associated with good treatment.

Regarding the explicit conceptualization, the words associated with good treatment refer to observable behaviors and, therefore, can be identified in the specific context in which they occur. Thus, concepts like ‘kindness’, ‘understanding’, or ‘listening’ contribute to a specific conceptualization of how one individual positively engages with another. (See Figure 8).

The relationship between the defining words obtained in the first hierarchy of the SAM set and the training cycle (pre-clinical and clinical) was then evaluated using Pearson’s chi-square test. Based on a bilateral contrast, it is evident that there is a statistically significant relationship between the defining words for good treatment and the training cycle, x^2^ (28.698) = *p* < 0.05, and a statistically significant relationship with the gender variable, x^2^ (41.158) = *p* < 0.05. In Table 4 and Table 5, the frequencies obtained for the training cycle and gender can be observed, and it is possible to recognize that the two words with the highest semantic weight (respect and empathy), which constitute the latent dimension, have higher frequencies than the words associated with the explicit dimension, for both cases.

## 4. Discussion

The educational environment requires special attention, as it provides a field of action from which the teaching–learning process is established. This field of action should aim not only at acquiring technical-clinical skills but also at internalizing a professional role. Modeling, as well as the educational relationship, is crucial for how a student perceives their formative experience, which is why the hidden curriculum plays a particular role in the training of health careers [6,7]. When situations of humiliation, intimidation, or discrimination arise, it is urgent to consider the consequences for the students, just as when positive behaviors that favor their proper development in the training process are made visible. In this scenario, the educational environment becomes a core element of the educational process that needs to be clarified in its multidimensionality, as evidenced in this study. The scope of the results obtained is descriptive and relational; therefore, we recommend positioning each of the meanings as guidelines for conceptualization rather than as saturated definitions of these terms.

Firstly, it is worth highlighting the semantic richness that emerges around mistreatment and good treatment, with more than 4000 words associated with each. This allows us to see at the outset that these terms are far from being simple or limited concepts, as they carry a highly polysemous character. In a certain way, the information code related to these terms is already loaded with ideas or emotions [46], which evokes participants to propose words as varied as those evidenced in this research. The delimitation that has been made depended on the semantic weight and hierarchy that the subjects granted them when mentioning them. In this sense, 14 primary defining words constituted the central core of the network. This systematization highlights a direct association with the subjects’ experience in the studied field, even though no specific situations of the formative context were specified in the data collection.

Secondly, it is necessary to note the dimensionality that emerges from the semantic cores, which operates from both a latent and explicit character. In the case of mistreatment, the latent meanings would be “aggression”, “abuse”, “violence”, “harm”, “discrimination”, “pain” and “power”. All these imply implicit forms of mistreatment manifestation, which cannot be visualized concretely, and we could assume that it transcends the subject-to-subject relationship unlike visible behaviors such as “humiliation”, “belittlement”, “hits”, “insults”, “harassment”, “mockery”, or “denigration”. This aligns with Galtung’s [25] proposal, which situates a direct sphere of violent behaviors as well as the legitimation of its use in latent terms such as cultural and structural. Now, it is important to specify that words with greater semantic weight could be considered central axes in the conceptualization of mistreatment, such as “aggression”, “abuse”, and “violence”. Thus, a preliminary definition of mistreatment would imply the interaction between latent and explicit actions, imposing on the other an experience of degradation, where violence, aggression, and abuse coexist in various forms of manifestation. Following the recommendations given by Hernández and Alvear [7], it is necessary to highlight the elements that bring out the multidimensionality of mistreatment, considering that there are already definitions include notions such as psychological violence, harassment, humiliation, defamation, disdain, aggression [24].

In the case of good treatment, the latent meanings evidenced were “respect”, “empathy”, “happiness”, “solidarity”, “love”, “endearment”, and “trust”. All of these denote areas of recognition and positive appreciation towards the other. These notions could constitute the antecedents of a culture that promotes well-being in educational training, which, as Olave et al. [33] state, depends on the ability of the subjects to establish empathetic, respectful, and caring relationships. On the other hand, the words with an explicit character were “kindness”, “understanding”, “tolerance”, “listening”, “help”, “support”, and “communication”. In all of them, it is possible to see forms of gentle treatment and consideration that facilitate the interaction of the subject with themselves and others. This aligns with the skills inherent in professionalism [38]. It is worth noting that the words with greater semantic weight and which could be considered central axes in the conceptualization of good treatment are “respect”, “empathy”, and “kindness”. Thus, a preliminary definition would imply considering the interaction of latent and explicit actions, founded on mutual recognition and reciprocal appreciation, where respect, empathy, and kindness coexist as priority realms.

Thirdly, it is worth mentioning that the preliminary conceptualizations proposed in this research allow us to visualize the breadth with which mistreatment and good treatment can be defined, depending mainly on certain academic or sociodemographic variables. The bivariate results showed that pre-clinical cycle students give more emphasis to concepts such as “aggression”, “abuse”, and “violence” than clinical cycle students. This could be consistent with what has been evidenced in Chile, as students are becoming increasingly aware of those situations that are not conducive to their training and were previously normalized [23]. This would explain the emphasis given to certain words, according to the training cycle.

In the case of good treatment, positive correlations were found in the two variables considered: training cycle and gender. Pre-clinical cycle students give more strength to words like “respect”, “understanding”, and “happiness” compared to clinical cycle students, who emphasize words like “empathy” and “kindness”. This aligns with the value given to positive relationships in their various forms of manifestation and, therefore, the need to implement them in training programs [50,51]. Furthermore, women tend to highlight words like “respect”, “empathy”, and “love”, which would constitute a relevant area for collaboration or, as López-Morales et al. [39] state, for professionalism.

The emphasis observed in the different meanings identified emerges from association networks. The semantic network technique offers this empirical means to access the cognitive organization that subjects have around a particular concept [52], through which different connotations could be observed in this instance. Now, it is important to emphasize that the recommendations that could offer clear conceptual definitions of mistreatment and good treatment would not only go in the order of promoting positive environments in health education but also in the official documentation of protocols to which training schools can adhere, such as the creation of policies or protocols associated with prevention. Moreover, among the actions recommended to address mistreatment, Olivares et al. [24] suggest having reliable complaint channels, mentoring, support networks, and guidance to the academic community on the types of mistreatments. For this, it is important to recognize the latent and explicit nature of behaviors that students may perceive in their formative experience. Ultimately, in terms of complaints, it is essential for educational institutions to generate resolutive actions [24]. Likewise, clear definitions of mistreatment and good treatment would allow for consensus on permissible and non-permissible behavioral manifestations within learning spaces, which would help generate greater clarity and promote the protection of iatrogenic experiences for students through the corresponding complaints and sanctions.

In relation to the above, it is worth mentioning some limitations of this study. The generalization of the findings may be limited since the study focused on specific universities in Chile, which may not reflect the full diversity of experiences in different educational and cultural contexts in other countries. This study did not examine some important variables that could influence the results, such as the specific institutional policies of the affiliated universities or the availability of psychological support and counseling for students, which could affect perceptions of mistreatment and good treatment reported.

Prevention and promotion must go hand in hand, as it is not possible to separate positive aspects from negative ones in training. These are realities that have been documented in scientific evidence, and therefore, we cannot think of good treatment in terms of promotion without thinking of mistreatment in terms of prevention. Considering this, as future lines of research, it is proposed to conduct co-creative design meetings between students and teachers to create decalogues on mistreatment and good treatment, so that they serve as a guide for various higher education institutions and allow for a contrast of the results found here. Additionally, it is proposed to investigate how specific institutional policies and psychological support programs influence the incidence and perceptions of mistreatment and good treatment. This could include evaluating the impact of different student support and counseling models. Finally, it is proposed to create predictive models that identify risk and protective factors associated with mistreatment and good treatment, allowing for earlier and more targeted interventions.

## 5. Conclusions

The educational environment is crucial in the teaching–learning process and significantly influences the development of ethical, empathetic, and effective health care professionals. Exploring mistreatment and good treatment in health careers impacts the quality of future professionals and patient well-being. Clear definitions of these concepts are vital for improving educational quality and underpinning effective interventions and policies. A safe and constructive learning environment fosters positive teacher–student relationships and supports student well-being. Additionally, standardizing evaluation instruments based on shared definitions enhances educational quality monitoring and aligns with the growing demand for student rights, promoting integrity and respect in educational institutions.

## Figures and Tables

**Table 1:** Description of the semantic network corresponding to the general SAM set for mistreatment.

	M	FREC	FMG
1. Aggression	2595	296	100%
2. Abuse	2520	279	97.1%
3. Violence	2326	254	89.6%
4. Humiliation	1990	233	76.6%
5. Harm	1624	188	62.5%
6. Belittlement	1103	142	42.5%
7. Hits	839	97	32.3%
8. Insults	757	101	28.9%
9. Discrimination	647	80	24.9%
10. Harassment	647	80	24.9%
11. Mockery	566	78	21.8%
12. Pain	564	70	21.7%
13. Denigration	564	67	21.7%
14. Power	480	59	18.4%

**Table 2:** Association between defining words for mistreatment and the training cycle of health science students.

Defining Words	Pre-Clinical	Clinical
1. Aggression	20.0%	16.8%
2. Abuse	23.8%	17.3%
3. Violence	16.6%	23.4%
4. Humiliation	4.9%	11.7%
5. Harm	9.8%	11.2%
6. Belittlement	3.0%	3.8%
7. Hits	8.7%	4.3%
8. Insults	1.1%	0.3%
9. Discrimination	2.6%	1.8%
10. Harassment	2.3%	0.8%
11. Mockery	0.0%	1.3%
12. Pain	2.6%	1.8%
13. Denigration	3.8%	2.5%
14. Power	0.8%	3.3%
Total frequencies	265	394

x^2^ = 35.858; *p* = 0.001.

**Table 3:** Description of the semantic network corresponding to the general SAM set for good treatment.

	M	FREC	FMG
1. Respect	4319	458	100%
2. Empathy	3861	435	89.3%
3. Kindness	1963	250	45.4%
4. Understanding	1158	146	26.8%
5. Happiness	596	75	13.7%
6. Tolerance	593	75	13.7%
7. Listening	563	75	13.0%
8. Solidarity	553	74	12.5%
9. Love	492	58	11.3%
10. Endearment	480	64	11.1%
11. Help	455	58	10.5%
12. Support	447	55	10.3%
13. Communication	436	54	10.0%
14. Trust	435	54	10.0%

**Table 4:** Association between defining words for good treatment and the training cycle of health science students.

Defining Words	Pre-Clinical	Clinical
1. Respect	55.3%	44.1%
2. Empathy	20.3%	31.3%
3. Kindness	2.8%	5.4%
4. Understanding	3.3%	3.0%
5. Happiness	3.3%	2.5%
6. Tolerance	1.6%	0.5%
7. Listening	0.4%	1.6%
8. Solidarity	0.8%	0.0%
9. Love	3.7%	4.4%
10. Endearment	1.2%	1.1%
11. Help	0.8%	1.4%
12. Support	2.4%	0.8%
13. Communication	2.8%	0.8%
14. Trust	1.2%	3.0%
Total frequencies	246	367

x^2^ = 28.698; *p* = 0.007.

**Table 5:** Association between defining words for good treatment and the gender of health science students.

	Man	Woman	Other
1. Respect	42.9%	51.2%	33.3%
2. Empathy	28.8%	26.3%	33.3%
3. Kindness	5.4%	4.0%	0%
4. Understanding	3.8%	2.6%	0%
5. Happiness	3.8%	2.3%	0%
6. Tolerance	0.5%	1.2%	0%
7. Listening	0.5%	1.2%	33.3%
8. Solidarity	0.5%	0.2%	0%
9. Love	3.3%	4.5%	0%
10. Endearment	1.1%	1.2%	0%
11. Help	2.2%	0.7%	0%
12. Support	2.7%	0.9%	0%
13. Communication	1.1%	1.9%	0%
14. Trust	3.3%	1.9%	0%
Total frequencies	184	426	3

x^2^ = 41.158; *p* = 0.030.

## Data Availability

Data are contained within the article.

## References

[1] Chacko K., Aagard E., Irby D. (2007). Teaching models for outpatient medicine. Clin. Teach..

[2] Ranjan N., Yousuf S., Tahseen M., Elhassan M., Saiful M., Faisal M., Van Mook W., Saeed M., Hameed H., Osman W. (2020). Preferred teaching styles of medical faculty: An international multi-center study. BMC Med. Educ..

[3] Dois A., Bravo P. (2024). Ejercicio docente y buen trato al usuario en el encuentro clínico de enfermería. Educ. Médica.

[4] Posada M., Vargas V., Orrego C., Cataño C., Vásquez E., Restrepo D. (2023). Educational environment and mental weelbeing of medical and surgical postgraduate residents in Medellin, Colombia. Rev. Colomb. Psiquiatr..

[5] Sattar K., Saiful M., Nor Arifin W., Azhar M., Zarawi M. (2023). A scoping review on the relationship between mental wellbeing and medical professionalism. Med. Educ. Online.

[6] Ortega J., Fasce E., Pérez C., Ibañez P., Márquez C., Parra P. (2014). Evaluación de componentes del currículum oculto en el pregrado de Medicina. Rev. Med. Chile.

[7] Hernández H., Alvear G. (2020). Violencia en la formación médica. Rev. Fac. Med..

[8] Association of American Medical Colleges (AAMC) (2020). Medical School Graduation Questionnaire: All Schools Report.

[9] Silver H. (1982). Medical students and medical school. JAMA.

[10] Naothavorn W., Puranitee P., Kaewpila W., Sumrithe S., Heeneman S., Van Mook W., Busari J. (2023). An exploratory university-based cross-sectional study of the prevalence and reporting of mistreatment and student-related factors among Thai medical students. BMC Med. Educ..

[11] Hayward L., Mott N., McKean E., Dossett L. (2023). Survey of student mistreatment experienced during the core clinical clerkships. Am. J. Surg..

[12] Dyrbye L., Satele D., West C. (2021). Association of Characteristics of the Learning Environment and US Medical Student Burnout, Empathy, and Career Regret. JAMA Netw. Open.

[13] Minton C., Birks M. (2019). “You can’t escape it”: Bullying experiences of New Zealand nursing students on clinical placement. Nurse Educ. Today.

[14] Shapiro J., Boyle M., McKenna L. (2018). Midwifery student reactions to workplace violence. Women Birth.

[15] Capper T., Muurlink O., Williamson M. (2021). Social culture and the bullying of midwifery students whilst on clinical placement: A qualitative descriptive exploration. Nurse Educ. Pract..

[16] Rowland M., Naidoo S., AbdulKadir R., Moraru R., Huang B., Pau A. (2010). Perceptions of intimidation and bullying in dental schools: A multi-national study. Int. Dent. J..

[17] Premadasa I., Wanigasooriya N., Thalib L., Ellepola A. (2011). Harassment of newly admitted undergraduates by senior students in a Faculty of Dentistry in Sri Lanka. Med. Teach..

[18] Lall M., Bilimoria K., Lu D., Zhan T., Barton M., Hu Y., Beeson M., Adams J., Nelson L., Baren J. (2021). Prevalence of Discrimination, Abuse, and Harassment in Emergency Medicine Residency Training in the US. JAMA Netw. Open.

[19] Bermeo J., Castaño-Castrillón J., López-Roman A., Téllez D., Toro-Chica S. (2016). Abuso académico a estudiantes de pregrado por parte de docentes de los programas de medicina de Manizales, Colombia. Rev. Fac. Med..

[20] Munayco-Guillén F., Cámara-Reyes A., Muñoz-Tafur J., Arroyo-Hernández H., Mejia C., Lem-Arce F., Miranda-Soberón U. (2016). Características del maltrato hacia estudiantes de medicina de una universidad pública del Perú. Rev. Peru. Med. Exp. Salud Publica.

[21] Mejia C., Quiñones-Laveriano D., Isela J., Aguirre-Valenzuela E., Heredia-Torres P., Miñan-Tapia A. (2018). Factores socioeducativos asociados a la percepción de maltrato en estudiantes de medicina peruanos. Educ. Médica Super..

[22] Real-Delor R., Acosta M., Aguilar E., Benítez L., Bordón L., Delgado L., Encina L., Escobeiro de la Cruz M., Maidana R., Quintana H. (2022). Maltrato a estudiantes de medicina del Paraguay en 2022: Encuesta multicéntrica. Med. Clín. Soc..

[23] Bastías-Vega N., Pérez-Villalobos C., Alvarado-Figueroa D., Schilling-Norman M., Espinoza-Riffo M., Parra-Ponce P., Matus-Betancourt O., Toirkens-Niklitschek J. (2021). Maltrato en el pregrado de la carrera de medicina. Rev. Med. Chile.

[24] Olivares S., Gómez J., Flores C., Castañeda A., Lizzeth M., Esperón R., Valdez-García J. (2021). Me preparo para prevenir la violencia y el acoso en estudiantes de medicina en México. Rev. Investig. Educ. Médica.

[25] Galtung J. (2003). Violencia Cultural.

[26] Singh T., Singh A. (2018). Abusive culture in medical education: Mentors must mend their ways. J. Anaesthesiol. Clin. Pharmacol..

[27] Pradhan A., Buery-Joyner S., Page-Ramsey S., Bliss S., Craig L., Everett E., Forstein D., Graziano S., Hopkins L., McKenzie M. (2019). To the point: Undergraduate medical education learner mistreatment issues on the learning environment in the United States. Am. J. Obs. Gynecol..

[28] Ortega J., Pérez C., Ortiz L., Fasce E., McColl P., Torres G., Wright A., Márquez C., Parra P. (2015). Estructura factorial de la escala DREEM en estudiantes de medicina chilenos. Rev. Med. Chile.

[29] Klein H., McCarthy S. (2022). Student wellness trends and interventions in medical education: A narrative review. Humanit. Soc. Sci. Commun..

[30] Lafuente J. (2019). El ambiente educativo en los contextos de formación médica. Educ. Médica.

[31] Schiwtz F., Torti J., Lingard L. (2023). What about Happiness? A clinical narrative review with implications for medical education. Perspect. Med. Educ..

[32] Hanco-Monroy D., Caballero-Apaza L., Abarca-Fernández D., Castagnetto J., Condori-Cardoza F., De Lama R., Carhuancho-Aguilar J., Gutierrez S., Gonzales M., Berduzco N. (2024). Medical professionalism and its association with dropout intention in peruvian medical students during the COVID-19 pandemic. Behav. Sci..

[33] Olave G., Pérez C., Fasce E., Ortiz L., Bastías N., Márquez C., Parra P., Ibañez P. (2016). Factores que afectan al ambiente educativo en la formación preclínica de medicina según sus docentes. Rev. Med. Chile.

[34] Sandoval S., Dorner A., Véliz A. (2017). Bienestar psicológico en estudiantes de carreras de la salud. Investig. Educ. Med..

[35] González-Contreras A., Pérez-Villalobos C., Hechenleitner M., Vaccarezza-Garrido G., Toirkens-Niklitschek J. (2019). Satisfacción académica y prácticas pedagógicas percibidas por estudiantes de salud de Chile. FEM.

[36] Bipin A., Singh H. (2019). Teaching models in the clinical years of medical education. Adv. Med. Educ. Pract..

[37] Mashauri H. (2023). Who should be a medical educator? Beyond knowledge and experience. Ann. Med. Surg..

[38] Delgado R., San-Martín M., Vivanco L. (2022). Role of empathy and lifelong learning abilities in physicians and nurses who work in direct contact with patients in adverse working conditions. Int. J. Environ. Res. Public Health.

[39] López-Morales H., Rivera-Díaz E., Ore-Zuñiga A., Vera-Portilla A., San-Martin M., Delgado R., Vivanco L. (2020). Positive impact of professionalism on the perception of global well-being. A study in healthcare professionals starting their first working experience in peruvian rural areas. Front. Public Health.

[40] Merrick D., Mbaki Y., Pratten M., Simpson T. (2021). Exploring wellbeing in first year medical students amidst a curriculum change. BMC Med. Educ..

[41] Suikkala A., Timonen L., Leino-Kilpi H., Katajisto J., Strandell-Laine C. (2021). Healthcare student-patient relationship and the quality of the clinical learning environment—A cross-sectional study. BMC Med. Educ..

[42] Exenberger S., Kunning M., Huber A., Prodinger W., Siller H., Medicus E., Brenner E., Schüßler G., Höfer S. (2021). Kommunikative un soziale Kompetenzen in Medizincurriculum der Medizinische Universität Innsbruck: Lernziele, Inhalt un Didaktik. GMS J. Med. Educ..

[43] Lind K., Osborne C., Badesch B., Blood A., Lowenstein S. (2019). Ending student mistreatment: Early successes and continuing challenges. Med. Educ. Online.

[44] Cushing R. (2024). Clinical Preceptor Development and the Benefit of Structured Teaching Techniques: A Scoping Review. J. Physician Assist. Educ..

[45] Bericat E. (1998). La Integración de Los Métodos Cuantitativo Y Cualitativo en la Investigación Social. Significado Y Medida.

[46] Valdez J. (1998). Las Redes Semánticas Naturales: Usos Y Aplicaciones en Psicología Social.

[47] Figueroa J., González E., Solís V. (1981). Una aproximación al problema del significado: Las redes semánticas. Rev. Latinoam. Psicol..

[48] Hinojosa G. (2008). El tratamiento estadístico de las redes semánticas naturales. Rev. Int. Cienc. Soc. Y Humanidades.

[49] De Souza L. (2019). Pesquisa como análise qualitativa de dados: Conhecendo a Análise Temática. Arq. Bras. Psicol. Río Jan..

[50] Jennings M., Slavin S. (2015). Resident wellness matters. Optimizing resident education and wellness through the learning environment. Acad. Med..

[51] Kienle R., Freytag J., Lück S., Eberz P., Langenbeck S., Sehy V., Hitzblech T. (2021). Das Kommunikationscurriculum im Modellstudiengang Medizin an der Charité—Universitaätsmedizin Berlin. GMS J. Med. Educ..

[52] Vásquez A., Rojo R., Navarro N. (2018). Concepción del docente motivador: Percepción de los estudiantes de una carrera de la salud. Investig. Educ. Med..

